# Quality of Life and Specific Difficulties of Children with Autism Spectrum Disorder Among Saudi Primary Caregivers

**DOI:** 10.3390/healthcare14172743

**Published:** 2026-08-28

**Authors:** Hanan Abd Elwahab Elsayed, Sultan Ahmed Alanazi, Manal Saleh Moustafa Saleh

**Affiliations:** 1 Assistance Medical Science Department, Applied College, University of Tabuk, Tabuk 71411, Saudi Arabia; 2 Department of Special Education, College of Education, University of Tabuk, Tabuk 71411, Saudi Arabia; 3 Department of Nursing Sciences, College of Applied Medical Science, Shaqra University, Shaqra 11961, Saudi Arabia

**Keywords:** quality of life, specific difficulties, autism spectrum disorder, Saudi primary caregivers

## Abstract

Background: The prevalence of autism spectrum disorder (ASD) is increasing globally and in Saudi Arabia (SA), although many cases remain undiagnosed. Caregivers of children with ASD are confronted by challenges, especially mothers as primary caregivers, who have higher levels of stress and burden of care due to autism symptoms and difficulties of children, which negatively affects their quality of life (QoL). Objective: The primary outcome of this study was to assess primary caregivers’ quality of life and their perceptions of child-related difficulties associated with autism spectrum disorder (ASD) using the Quality of Life in Autism (QoLA) questionnaire. The secondary outcome was to examine the correlation between these two QoLA domains and identify the factors independently associated with each outcome Material and Methods: A convenience sample (247) of Saudi primary caregivers, primarily mothers, was taken from autism primary schools in Tabuk, Riyadh, Qassim, Hail, Jazan & Najran cities in SA by distributing an online questionnaire, which was composed of three parts, was used for data collection; Part I included demographic and clinical data of primary caregivers. Part II: The child’s personal characteristics. Part III: QoLA Questionnaire that measures the crucial aspects of living with autistic children and is composed of two parts (A and B). Data analysis was performed using the Statistical Package for the Social Sciences (SPSS Inc., Chicago, IL, USA), version 23. Results: More than half of the caregivers (50.2%) had a moderate level of QoL. Regarding caregivers’ perceptions of child-related difficulties, 70.9% reported high levels of difficulties, and 7.2% reported low difficulties. Quality of life was significantly associated with maternal age, educational level, number of children. The study’s univariate regression and multivariate regression analysis revealed that, QoL score had a significant negative association with maternal ages, university school and secondary school, working, and those who had medical diseases. In contrast, QoL level had a significant positive association with mothers who received training, had another person helped in caring for the child and who had enough income. Conclusions: Mothers of autistic children had poorer QOL and perceived more difficulties in caring for their children. Thus, educating sessions for mothers about dealing with their autistic children, psychological and financial support services are needed, which in turn improve their QoL.

## 1. Background

Autism spectrum disorder (ASD) is a prevalent neurodevelopmental disorder characterized by permanent constraints in interaction and communication at the social level, characterized by repetitive patterns of activities, behavior, and interests that differ in severity. Hereditary, parental, and environmental factors are considered among the major contributors to the development of autism spectrum disorder (ASD) [1]. In addition to the core symptoms of ASD, children with ASD are at increased risk of developing comorbid medical conditions, such as epilepsy and gastrointestinal disorders [2]. Globally, ASD is estimated to affect approximately one in every 44 children [3]. Epidemiological studies have reported considerable variation in the prevalence of ASD across the Gulf region, with estimates ranging from 1.4 to 29 per 10,000 individuals [4]. In Saudi Arabia, epidemiological data remain limited; however, the available evidence estimates the prevalence of ASD at approximately 18 per 10,000 individuals [5]. Autistic children experience challenges across all subsystems of language, both orally and in writing. Furthermore, given these communication challenges, some children may be very verbal, while others may not. Caring for an autistic child is considered very challenging for parents, which significantly affects their emotional well-being and quality of life (QoL) due to challenges during their childhood years, in their school years, and generally in life [6,7]. Some studies in Saudi Arabia stated that caregivers of autistic children had higher levels of anxiety and depression than parents of developing non-autistic children due to the severity of symptoms, comorbidities, and difficulties in autistic children. Consequently, lower levels of QoL were reported among caregivers of autistic children in all physical, psychological, social, and environmental domains compared to caregivers of normal children [8,9].

Caregivers of autistic children suffer a negative QoL due to the active nature of the challenges that they confront. When the child is initially diagnosed with ASD, caregivers must learn to accept, familiarize themselves with, and manage new needs and information [10]. Between the ages of 4 and 8 years, caregivers face additional comorbidities alongside emotional or behavioral symptoms [11]. Caregivers’ QoL is influenced by severe main features of ASD, especially abnormal behaviors such as high activity levels, oppositional compliance, attitude problems, anxiety, overall delay in development, and impaired daily living activities [12]. A study conducted in Tabuk City, on one hundred caregivers of children with autism, found that psychological and environmental domains of QoL are most strongly affected, and socio-demographic factors play significant roles in shaping QoL consequences [13].

Furthermore, in Arar City, a study on eighty-four parents of autism children with autism revealed that nearly two-thirds of caregivers experienced impaired QoL [14]. Generally, caregivers of autistic children tend to have lower total QoL scores in most domains, suggesting an increased burden of caring for children with ASD. A cross-sectional study in Jeddah assessing 812 caregivers across Saudi provinces found that caregivers of ASD children scored significantly lower across most QoL domains compared with caregivers of normal children [15].

Financial difficulties are the primary concern for caregivers due to the intensity and cost of speech-language training; consequently, some caregivers are forced to stop their children’s language training [16]. In SA, culture and customs, and the exhaustive training needed for active intervention, more than thirty hours per week, are considered other challenges for caregivers. Although Saudi families with autistic children receive free services and funding from the government, these services are not sufficient to satisfy all children’s needs, and most of them do not have access to these services [17].

At present, the Saudi government, as part of the QoL program under Saudi Vision 2030, has launched large-scale initiatives to improve the QoL of individuals with diverse health conditions and careers, especially disabled people and autistic children in SA [18]. Although previous Saudi studies have evaluated caregivers’ quality of life using generic quality-of-life instruments, evidence based on the autism-specific Quality of Life in Autism (QoLA) questionnaire remains limited. Moreover, few studies have simultaneously assessed caregivers’ quality of life and their perceptions of child-related difficulties using an ASD-specific instrument. Understanding how Saudi primary caregivers experience these challenges within their cultural and healthcare context is essential for informing supportive interventions, healthcare planning, and policy development. Therefore, the present study used the validated Quality of Life in Autism (QoLA) questionnaire, an ASD-specific instrument comprising two subscales: Part A (QoLA-A), which assesses caregivers’ perceived quality of life, and Part B (QoLA-B), which evaluates caregivers’ perceptions of child-related difficulties associated with ASD [19]. Accordingly, this study aimed to investigate the quality of life of Saudi primary caregivers of children with ASD and their perceptions of autism-related child difficulties using the QoLA questionnaire.

## 2. Materials and Methods

### 2.1. Study Design

A cross-sectional design was used in this study.

### 2.2. Setting

The present study data were collected from autism primary schools in Tabuk, Riyadh, Qassim, Hail, Jazan & Najran cities in SA by distributing an online questionnaire to caregivers of children with ASD who were registered at the schools via social media or caregiver groups.

### 2.3. Sample Size

The minimum required sample size was calculated using G*Power version 3.1 [20]. Assuming a significance level (α) of 0.05, a statistical power of 80%, and a medium effect size, the minimum required sample size was estimated to be 197 participants. To compensate for potential incomplete questionnaires or ineligible responses, we planned to recruit approximately 25% more participants, resulting in a recruitment target of 247 caregivers. After data screening, all completed questionnaires met the eligibility criteria; therefore, no questionnaires were excluded, and all 247 participants were included in the final analysis. Eligible participants were primary caregivers (male or female) of children diagnosed with ASD. However, all respondents who completed the questionnaire were mothers. Therefore, the findings reported in this study reflect maternal primary caregivers, reflecting the caregiving pattern commonly observed in Saudi families.

### 2.4. Study Measures and Data Collection

Data were collected between April 2025 and November 2025 using an online questionnaire distributed via email and social media across six regions of Saudi Arabia (Tabuk, Riyadh, Qassim, Jazan, Hail, and Najran). Eligible participants were recruited through autism primary schools, where designated data collectors in each region facilitated dissemination of the survey link to primary caregivers of children with ASD. Participation was voluntary, and caregivers were informed about the study objectives, confidentiality of their responses, and their right to withdraw at any time before providing electronic informed consent. Although both male and female primary caregivers were eligible to participate, the study sample consisted predominantly of mothers, reflecting the caregiving pattern commonly observed in Saudi families. The questionnaires were composed of three parts:

Part I: Demographic and clinical data of primary caregivers, such as age, social status, educational level, income, occupation, number of children, children with special needs, medical disease, training sessions, other person helped in caring for the child, and social or psychological support group.

Part II: The child’s personal characteristics, such as age, sex, other comorbidities, taking drugs, follow-up with specialists, and receiving special training courses.

Part III: QoLA Questionnaire that measures all the critical aspects of living with autistic children and has two parts (A and B) with internal consistency of 0.94 and 0.92 for part A and part B, respectively [19].

Part A is the QoL subscale, comprising 28 questions that measure QoL as perceived by caregivers. For example, “I am satisfied with my general health,” “I am satisfied with my achievements,” and other items that had reversed scores on a five-point Likert scale, such as “I feel stressed” and “Health problems stop me from doing things that I want to do.” Answers on the Likert scale range from one, which means not very much, to five, which means very much. Hence, total scores on part A (QoLA) ranged from 28 to 140. As scores increased, better QoL was perceived. This part covers many aspects like financial condition, health, self-satisfaction, community support, and adverse psychological effects.

Part B consists of 20 items that assess caregivers’ perceptions of the extent to which ASD-related behaviors are problematic in everyday life. Example items include “Has Friends,” “Understands others’ feelings,” “Understands the basics of social interaction,” and “Performs daily living tasks independently.” Each item is rated on a five-point Likert scale ranging from 1 (very much of a problem for me) to 5 (not much of a problem for me). Therefore, total Part B scores range from 20 to 100, with higher scores indicating fewer perceived child-related difficulties and lower caregiving burden associated with ASD-related behaviors.

Therefore, the researchers scored Parts A and B independently and represented them as separate subscales within the questionnaire.

For descriptive purposes, caregivers’ quality of life and perceived child-related difficulties were categorized into three levels based on the percentage of the maximum possible score. Scores of <60% were classified as low, scores of 60% to <75% as moderate, and scores of ≥75% as high.

Previous validation studies reported Cronbach’s alpha coefficients of 0.93 for Part A and 0.91 for Part B [21]. The data collection period extended from April 2025 to November 2025. The primary outcome of this study was to assess primary caregivers’ quality of life and their perceptions of child-related difficulties associated with autism spectrum disorder (ASD) using the Quality of Life in Autism (QoLA) questionnaire. The secondary outcome was to examine the correlation between these two QoLA domains and identify the factors independently associated with each outcome.

### 2.5. Ethical Considerations

The Deanship of Scientific Research at the University of Tabuk accepted the project proposal (No. S-2024-0058, 18 February 2025). After that, Ethical clearance was obtained from Shaqra University’s standing committee of research ethics (ERC_SU_S_202500124 in 17 March 2025), upon determining that the research met its requirements and complied with the rules of the National Committee of Bioethics. Participation was voluntary, and informed consent was obtained after stressing the study’s purposes, confidentiality, secure data storage, the absence of risk of harm, and participants’ right to withdraw at any time. The researchers obtained additional approvals from the previously mentioned settings before data collection. In addition, consent was obtained by asking to confirm that they agreed to complete the questionnaire by marking a “Yes, I agree to participate.” Participants gave their informed consent under the criteria outlined in the Declaration of Helsinki.

### 2.6. Statistical Methods

Data were analyzed using IBM SPSS Statistics for Windows, Version 23.0 (IBM Corp., Armonk, NY, USA). Descriptive statistics were used to summarize caregivers’ sociodemographic characteristics and children’s clinical characteristics and were presented as frequencies, percentages, means, and standard deviations. Differences in Quality of Life in Autism (QoLA) Part A and Part B scores according to participants’ characteristics were examined using the paired-samples *t*-test and one-way analysis of variance (ANOVA), as appropriate. Pearson’s correlation coefficient (r) was used to assess the relationship between caregivers’ QoL scores and child-related difficulties. Before conducting the inferential analyses, the assumptions of normality, homogeneity of variances, linearity, and independence were assessed and found to be satisfactory.

Univariate linear regression analysis was performed to examine the association between each independent variable and QoLA Part A and Part B scores. Continuous variables included caregivers’ age and number of children, whereas categorical variables included marital status, educational level, occupation, monthly family income, and other sociodemographic characteristics. For categorical variables, the first category was used as the reference category. Given the exploratory nature of this study, univariate regression analysis was used to evaluate the association of each independent variable with caregivers’ quality of life separately. Therefore, the findings should be interpreted as unadjusted associations rather than independent effects. Multivariable regression analyses were performed to account for potential confounding variables. A *p*-value of <0.05 was considered statistically significant.

## 3. Results

A total of 247 primary caregivers completed the questionnaire. All respondents were mothers. The socio-demographic characteristics of the participants are presented in Table 1. Among the participating primary caregivers, the majority were mothers who were housewives (93.1%). More than half of them were aged older than 40 years (50.6%), married (85.8%), had a university education (42.9%), and had insufficient income (42.1%). Moreover, more than half of mothers (60.6%) had 4–6 children, (24.3%) were from Tabuk city, had another child with special needs (28.3%), were not receiving any training sessions (78.5%), and (70.9%) were free from any medical diseases. In addition, more than three-quarters (77.7%) do not have anyone to help them with caring for the child. However, 72.1% did not participate in a social or psychological support group.

Table 2 reveals the personal characteristics of children with ASD. Most of the children (78.9%) were six to less than twelve years old, with a (Mean ± SD) of 9.00 ± 3.37, and did not have other comorbidities (71.3). Furthermore, participants underwent drug therapy (64%), followed by a follow-up with specialists (64.4%). More than half of them (50.6%) received special training sessions to improve speech and communication skills.

**Figure 1:**
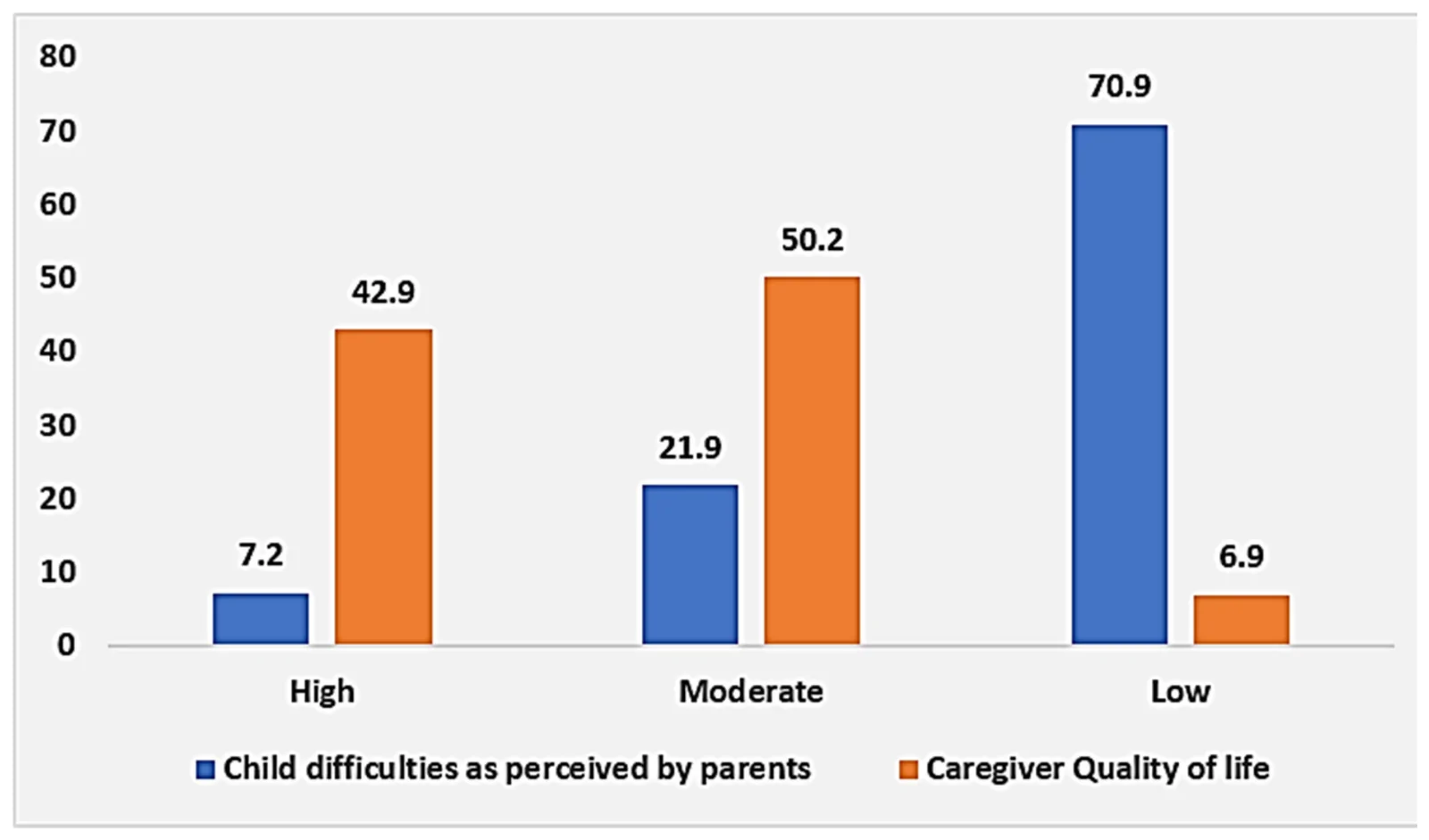
Total QoL (section A) and child difficulties (section B) scores as perceived by caregivers in the QoLA questionnaire. High perceived QoLA-B score (fewer difficulties); Low score (greater difficulties).

Figure 1 represents the total QoL (section A) and child difficulties (section B) as perceived by caregivers in the QoLA questionnaire. According to the QoLA Part B scoring, higher scores indicate fewer perceived child-related difficulties. Accordingly, 70.9% of caregivers reported a high level of perceived child-related difficulties, whereas only 7.2% reported a low level of perceived child-related difficulties. In terms of QoLA Part A, 50.2% of caregivers had a moderate QoL, while only 6.9% had a low QoL.

Table 3 represents the factors significantly associated with section A (QoLA) questionnaire and section B (QoLA). The QoL scores are higher in working mothers (*p* = 0.000), those who had only one child of special needs (*p* = 0.005), received training sessions (*p* = 0.000), not having medical diseases (*p* = 0.000), had another person helped them in caring of their children (*p* = 0.000), and enrolled in social or psychological support groups through media (*p* = 0.000). Regarding section B, perceived difficulties when caring for autistic children increased among married mothers (*p* = 0.000), housewives (*p* = 0.000), having medical diseases (*p* = 0.042), not having another person help in caring for the child (*p* = 0.042), and not having a social or psychological support group (*p* = 0.031).

Table 4 represents other factors significantly associated with section A (QoLA) questionnaire and section B (QoLA). As observed, QoL scores were significantly associated with mothers aged 20 to less than 30 years old (*p* = 0.01), reading and writing (*p* = 0.000), and having 4–6 children (*p* = 0.028). On the other hand, section B, perception of difficulties, was significantly associated with mothers aged 30 to 39 years (*p* = 0.000) and with mothers having 1–3 children (*p* = 0.000).

Table 5 uses univariate and multivariate regression analyses to investigate the associated factors with mothers’ perceptions of QoL (section A). In the univariate regression analyses, the QoL score had a significant negative association with maternal ages 30–<40 and 40+ (*p* < 0.001), university school and secondary school (*p* < 0.001), working (Estimate = −10.761, *p* < 0.001), and those with two children with special needs (Estimate = −3.557, *p* = 0.005). In contrast, QoL level had a significant positive association with mothers who had 4–6 children (*p* = 0.008), received training (*p* < 0.001), and had another person help in caring for the child (*p* < 0.001). In multivariate regression analyses QoL score had a significant negative association with maternal ages 30–<40 and 40+ (*p* < 0.001), university school and secondary school (*p* < 0.001), those with two children with special needs (Estimate = −21.023, *p* < 0.001) had medical disease (Estimate = −2.797, *p* < 0.001), On the other hand, QoL level had a significant positive association with mothers who had enough income (*p* < 0.001), had 4–6 and >6 children (*p* = <0.001).

Table 6 uses univariate and multivariate regression analyses to investigate the associated factors with mothers’ perceptions of child-related difficulties of section B (QoLA). In the univariate regression analyses, child-related difficulties score had a significant negative association with maternal ages 40+ (*p* = 0.009), university school (*p* = 0.046), and having enough income (Estimate = −4.577, *p* = 0.005). In contrast, child-related difficulties of section B (QoLA had a significant positive association with divorced or widowed (*p* < 0.001), working (*p* < 0.001), number of children 4–6 years (*p* < 0.001), and those who had medical disease (*p* = 0.042), and had another person helped in caring for the child (*p* < 0.001), mothers who had 4–6 children (*p* = 0.008). In multivariate regression analyses, child-related difficulties of section B (QoLA had a significant negative association with maternal ages 30–<40 and 40+ (*p* < 0.001), having enough income (*p* < 0.001), and receiving training (*p* < 0.001). On the other hand, child-related difficulties of section B (QoLA) had a significant positive association with divorced or widowed (*p* < 0.001), working (*p* < 0.001), number of children 4–6 and >6 years (*p* < 0.001), and those who had medical disease (*p* < 0.001), had another person helped in caring for the child (*p* < 0.001).

As shown in Table 7, the total caregivers’ QoL score (section A) had a significant negative correlation with the total child difficulties score (section B) (r = −0.415, *p* = 0.000).

## 4. Discussion

Although this study targeted primary caregivers of children with ASD, all respondents were mothers. Therefore, the findings should be interpreted as reflecting the experiences of maternal primary caregivers. Females with autistic children often face difficulties as they are the primary source of support for their children throughout their lives [22]. Parental QoL is negatively affected by raising an autistic child compared to raising normal children or children with chronic diseases. Several factors affected parental QoL, including socio-demographic and socioeconomic factors, as well as characteristics of autistic children [23,24]. Yet limited research has focused on evaluating the QoL of caregivers of autistic children in Saudi Arabia and their difficulties using the QoLA questionnaire. Hence, we presented the most comprehensive results that address QoL and specific difficulties of children with ASD among Saudi primary caregivers and related factors that affect them using more specific tools for autistic children.

Our current study revealed that more than half of the mothers were above 40 years old, married, housewives, had an insufficient income, and had 4–6 children. Furthermore, most mothers were not included in any social or psychological support groups and did not have anyone assist them with childcare. In addition, most of the autistic children received drug therapy, and more than half of them received training sessions to improve speech and communication skills. These characteristics highlight that those mothers of autistic children face multiple burdens in their daily lives that affect their QoL. Furthermore, our results showed that more than two-thirds of caregivers reported low or moderate QoL, which remained lower than that of other parents. This finding is consistent with previous findings from different countries that caregivers of autistic children showed significantly lower QoL in ASD-related QoL [25,26]. Similarly, a Saudi study across 13 provinces for autistic children used an online questionnaire derived from RAND SF-36. Found lower overall QoL scores in most areas except for one domain, pointing to an increased burden of caring for autistic children. Another study in Arar, SA, using the same measure, found impaired QoL for less than two-thirds of the eighty-four caregivers of autistic children. In addition, another study on a sample of 376 parents of children with ASD in SA detected lower QoL for mothers. It stressed the importance of modifying interventions that emphasize QoL for caregivers of children with autism [14,15,27].

Our study demonstrated that more than two-thirds of caregivers reported significant difficulties in caring for their children (QoLA Part B). This finding aligned with an analysis of 7 countries on Australian, Hungarian, Malaysian, Romanian, Singaporean, Spanish, and British autistic children that addresses QoL from a transcultural perspective. Reported significant differences in the mean QoLA Part B scores across the countries, of which the child’s age was statistically significant among other variables, indicate that the caregivers’ perception of their child’s ASD-specific difficulties is the same, irrespective of their cultures, as the Australian cohort of parents was the least affected by their child’s ASD-specific difficulties (Part B) [28]. In addition, another Saudi study in Medina detected that the mothers of autistic children perceived substantially lower sleep quality and psychological challenges [29]. These findings are the same as another study showing that mothers of children with ASD experience significantly lower QoL compared with mothers of normal children due to the stress of caregiving burden, behavioral difficulties, and limited social support [14]. This finding contradicts a study conducted in Qatar, which found no statistically significant differences in QOL domains between caregiving for children with ASDs and for children without ASDs, except in the general health domain. This contradiction can be explained by Qatar’s smaller population, which improves access to healthcare services and meets the needs of children with ASD and their caregivers, resulting in improved QOL [30].

Our study found that QoL scores were significantly associated with maternal age, education, number of children, and having only one child with special needs. This means that younger mothers and those with fewer children had better QoL than older mothers and those with larger families. This is consistent with a previous Saudi study that found age was statistically significant across most domains and detected a decline in QOL scores among older caregivers [15]. Also, other studies first pointed out that having more than one child diagnosed with ASD hurts parents’ QoL and detected a significant and negative relationship between the number of children and the QoL of caregivers with autistic children [31]. Second, a review of parental QoL of ASD children found that QoL level was lower in parents of children with ASD; parental education was a protective factor for their QoL, supporting early diagnosis and intervention, giving special attention to low-income families [32]. Third, a Saudi study in Jeddah found that QoL was significantly affected by social status and parents’ educational achievement [33]. Previous study reported that family size was significantly associated with QoL [26].

Additionally, in our study, significantly associated factors with QoL included working mothers who received training sessions, did not have medical conditions, had another person help them with caring for their children, and enrolled in a social or psychological support group through the media. These results are consistent with several previous studies. First, a study demonstrated that support groups are vital for caregivers and other family members of children with ASD to acquire proper emotional support and relieve stress, which are seriously needed for their well-being through professionals such as psychologists and special educators [34]. Second, a study in Jordan found that parents of children with ASD had poor QoL in all domains compared with parents of children with typical development. Factors such as educational level and employment status may affect QoL [35]. Contrary to our study, this study found that employed mothers had significantly higher QoL than housewives [36]. This variation may be due to differences in economic characteristics between the studies’ participants and our participants, who considered mothers a primary source of income for their families to meet their children’s needs, resulting in low socioeconomic status and inadequate living conditions. This differs in the SA, where men are the primary source of income and also receive government assistance. But in other countries, the lack of employment for women negatively impacts the family’s ability to meet their children’s needs and, consequently, their QoL.

The study’s univariate regression and multivariate regression analysis revealed that QoL score had a significant negative association with maternal ages 30–<40 and 40+, university school and secondary school, working, and those who had medical diseases. In contrast, QoL level had a significant positive association with mothers who received training, had another person help in caring for the child, and mothers who had sufficient income. Similarly, a Malaysian study found a positive association between attending more caregiver training sessions and QoL compared to those who attended only one session, the presence of a support group on Facebook, the presence of siblings or a person who helps with child care, the caregiver having asthma, and educational status [37]. In addition, in another study, regression analysis revealed that widowed or divorced mothers, occupation, and lower academic achievement predicted lower quality of life [38]. In a univariate analysis of another study in Eastern China, caregivers of children with high education, high household income, and no chronic illness had significantly higher QoL [39]. The regression model of predictors of QOLA-A in another study on Hungarian parents identified family income, social support, and parental age as significant predictors, suggesting that older parents have poorer QoL [40]. Similarly, a descriptive observational study in Indonesia was conducted by Samadi et al. (2025) [41]. A regression model showed that a higher quality of life was associated with parents’ approval of their health, support from family or a special person, and education level. However, the same study contradicts ours and found that parental age, income, and the number of children did not affect QoL ratings. In addition, a study by Rezq et al. (2025) [42] in SA. Found that family incomes were significantly positive associated factors of QoL; however, educational level was not associated with QoL scores. Alhazmi et al. (2023) found in their study in SA that educational level was a predictor of better QoL [43]. On the contrary, the previously mentioned study found no correlation between the number of siblings and QoL [40]. This may be attributed to differences in economic and social characteristics between the Saudi participants in our study and those in other studies.

A significant negative correlation between total caregivers’ QoL and total child difficulties score was detected in our study. This means increasing difficulties and severity of ASD symptoms are correlated with caregiver burden and lower QoL. This was congruent with Patel et al. (2022) [44]. The first integrative review study retrieved 15 related articles, which found that the most significant factors affecting parental QOL were the severity of the diagnosis of ASD for the child, and the second study detected a positive correlation between the severity of autism and burden of care in caregivers and a negative correlation between the severity of symptoms and the QoL in caregivers of ASD children [32]. Furthermore, another review study showed that the severity of symptoms of ASD in children was significantly positively correlated with parents’ symptoms, such as anxiety and depression, and negatively correlated with QoL of parents across all domains [45]. These findings shed light on the importance of evaluating parental QoL of ASD children, difficulties they faced in caring for them, and addressing the major factors that enhance their QoL. Through our study, significant areas for improvement will be identified, helping policymakers design and implement appropriate strategies by providing evidence-based school services that support parents in managing their children’s difficulties and improving their QoL.

## 5. Conclusions

Based on the results in this study, most mothers of our sample were aged, housewives, had insufficient income, and had 4–6 children. Furthermore, most mothers were not included in any social or psychological support groups, did not have anyone assist them with childcare, and did not receive any childcare training. In addition, most of the autistic children had other comorbidities and received drug therapy. These characteristics highlight that those mothers of autistic children face multiple burdens in their daily lives that affect their QoL. More than half of mothers reported moderate QoL and significant difficulties in caring for their children. Numerous factors that positively or negatively affect mothers’ QoL and their perceptions of their children’s problems were identified. QoL scores were significantly associated with maternal age, education, number of children, and having only one child with special needs.

Additionally, in our study, factors related to QoL were being a housewife mother, having received training sessions, having medical conditions, having another person help them with caring for their children, and being enrolled in a social or psychological support group through the media. Overall, the analysis indicates that younger age, being a housewife, higher educational level, absence of medical conditions, higher income, having another person help in caring for the child, and receiving training were significant positive associated factors of QoL. A significant negative correlation between total caregivers’ QoL and total child difficulties was detected. These previous factors can be used to reduce the burden of caring for ASD children with ASD and enhance caregivers’ QoL.

## 6. Strengths, Limitations, and Recommendations

A major strength of this study is that it included caregivers from autism primary schools across several regions of Saudi Arabia and employed the validated Quality of Life in Autism (QoLA) questionnaire to assess caregivers’ quality of life. In addition, the study examined several caregiver- and child-related factors associated with caregivers’ quality of life and perceived child-related difficulties.

The findings of this study should be interpreted within the context of the study design. First, the cross-sectional design precludes establishing causal relationships between the study variables. Second, participants were recruited using convenience sampling through autism schools, social media platforms, and caregiver groups, which may limit the generalizability of the findings. Third, data were collected using self-reported questionnaires, making the findings subject to response bias. Furthermore, although both male and female primary caregivers were eligible to participate, the study sample consisted predominantly of mothers; therefore, the findings mainly reflect maternal caregiving experiences. Finally, univariate regression analyses were conducted to explore the associations between individual variables and caregivers’ quality of life. Future studies may benefit from applying multivariable regression models to further evaluate these associations while accounting for potential confounding variables.

Future research should include caregivers of adolescents and adults with ASD, recruit more diverse caregiver groups, and incorporate more comprehensive clinical assessments of children with ASD to better understand factors associated with caregivers’ quality of life. Longitudinal studies are also warranted to examine changes in caregivers’ quality of life over time. In addition, developing and evaluating educational, psychological, and social support programs for caregivers may contribute to improving their quality of life.

## Figures and Tables

**Table 1:** Sociodemographic and clinical characteristics of primary caregivers (*n* = 247).

	No.	%
Age/years		
20–<30	35	14.2
30–<40	87	35.2
40+	125	50.6
Min–max	20–50	
Mean ± SD	41.33 ± 5.91	
Marital Status		
Married	212	85.8
Divorced/Widowed	35	14.2
Education		
Read and write	54	21.9
Secondary school	87	35.2
University Education	106	42.9
Income		
Not enough	104	42.1
Enough	143	57.9
Occupation		
Housewife	230	93.1
Working	17	6.9
No. of children		
1–3	87	35.2
4–6	125	50.6
>6	35	14.2
Region of residency		
Tabuk	60	24.3
Riyadh	55	22.3
Qassim	40	16.1
Jazan	33	13.3
Najran.	31	12.6
Hail	28	11.3
Children with special needs		
One	177	71.7
Two	70	28.3
Caregiver training		
No	194	78.5
Yes	53	21.5
Medical diseases		
No	175	70.9
Yes	72	29.1
Another person helped in caring for the child		
No	192	77.7
Yes	55	22.3
Social or psychological support group		
No	178	72.1
Yes	69	27.9

**Table 2:** The ASD children’s personal characteristics (*n* = 247).

	No.	%
Age/years		
6–<12	195	78.9
12+	52	21.1
Min–max	6–14	
Mean ± SD	9.00 ± 3.37	
Sex		
Male	187	75.7
Female	60	24.3
Other comorbidity		
No	176	71.3
Yes	71	28.7
Taking drug		
No	89	36.0
Yes	158	64.0
Follow up with specialists		
No	88	35.6
Yes	159	64.4
Receive special training		
No	122	49.4
Yes	125	50.6

**Table 3:** Comparison of the section A and section B scores in the (QoLA) questionnaire according to the variables in independent groups using the *t*-test.

	Section A		*t* Test	*p*-Value	Section B		*t* Test	*p*-Value
	Mean	±SD			Mean	±SD		
Marital status								
Married	57.43	9.722	1.793	0.074	89.51	10.897	9.290	0.000 **
Divorced/Widowed	108.17	11.663			54.48	0.5070		
Income								
Not enough	55.71	12.22	1.946	0.053	94.80	17.631	2.819	0.002
Enough	57.97	5.635			90.23	6.9502		
Occupation								
Housewife	57.76	8.959	4.943	0.000 **	91.13	12.649	4.836	0.000 **
Working	106.00	10.463			47.00	6.86		
Children with special needs								
One	58.02	9.716	2.818	0.005 **	92.51	13.824	0.696	0.487
Two	54.47	6.557			91.25	9.6469		
Caregiver training								
No	55.92	9.948	3.716	0.000 **	92.80	14.120	1.538	0.125
Yes	61.01	0.8201			89.77	4.9522		
Medical diseases								
No	60.59	4.704			91.09	14.510	2.048	0.042 *
Yes	55.54	9.984	4.104	0.000 **	94.73	6.3089		
Another person helped in caring for the child.								
No	55.19	9.420	6.379	0.000 **	95.43	4.9805	2.175	0.031 *
Yes	63.40	2.670			91.21	14.112		
Social or psychological support group								
No	52.15	11.36	5.556	0.000 **	96.55	10.409	10.420	0.000 **
Yes	58.90	7.195				80.81	11.267	

** Highly statistically significant *p* < 0.01. * Statistically significant *p* < 0.05.

**Table 4:** Comparison of the section A and section B scores in the (QoLA) questionnaire according to the variables in independent groups using the one-way ANOVA test.

	Section A		F Test	*p*-Value	Section B		F Test	*p*-Value
	Mean	±SD			Mean	±SD		
**Age**								
20–<30	61.28	10.14	4.674	0.010 *	95.08	3.042	9.138	0.000 **
30–<40	56.48	5.440			95.74	14.58		
40+	56.20	10.40			88.84	12.29		
**Education**								
Read and write	64.38	2.10	110.70	0.000 **	94.79	5.040	2.028	0.134
Secondary school	48.87	7.60			90.36	18.99		
University Education	59.95	7.16			92.28	8.229		
**No. of children**								
1–3	55.06	12.96	3.635	0.028 *	87.93	14.898	16.120	0.000 **
4–6	58.44	5.25			96.45	10.957		
>6	56.80	7.09			87.31	6.592		

** Highly statistically significant *p* < 0.01. * Statistically significant *p* < 0.05.

**Table 5:** Univariate and multivariate regression analyses of socio-demographic variables associated with the section A (QoLA) as perceived by caregivers.

		Univariate Regression Analysis				Multivariate Regression Analysis		
Predictors	Estimate	95% Confidence Interval		*p*-Value	Estimate	95% Confidence Interval		*p*-Value
		Lower Bound	Upper Bound			Lower Bound	Upper Bound	
Age								
30–<40 vs. 20–<30	−4.803	−8.325	−1.281	0.008	−2.246	−2.411	−2.080	<0.001
40+ vs. 20–<30	−5.086	−8.451	−1.721	0.003	−8.100	−8.316	−7.884	<0.001
Marital Status								
Divorced/Widowed—married	−2.953	−6.196	0.290	0.074	-	-	-	-
Education								
University school—Read and write	−15.515	−17.764	−13.266	<0.001	−21.536	−21.730	−21.342	<0.001
Secondary school—Read and write	−4.436	−6.607	−2.266	<0.001	−6.728	−6.913	−6.543	<0.001
Income								
Enough—Not enough	2.260	−0.028	4.549	0.063	7.987	7.784	8.190	<0.001
Occupation								
Working—housewife	−10.761	−15.049	−6.472	<0.001	-	-	-	-
No. of children								
4–6 vs. 1–3	3.371	0.904	5.838	0.008	0.828	0.669	0.987	<0.001
>6 vs. 1–3	1.731	−1.806	5.268	0.336	15.285	15.025	15.546	<0.001
Children with special needs								
Two—one	−3.557	−6.043	−1.070	0.005	−21.244	−21.466	−21.023	<0.001
Caregiver training								
Yes—no	5.091	2.393	7.789	<0.001	-	-	-	-
Medical diseases								
Yes—no	1.953	6.196	3.290	0.074	−2.987	−3.177	−2.797	<0.001
Another person helped in caring for the child.								
Yes—no	8.207	5.673	10.741	<0.001	-	-	-	-

This parameter is set to zero because it is redundant. highly statistically significant *p* < 0.01 statistically significant *p* < 0.05.

**Table 6:** Univariate and multivariate regression analyses of socio-demographic variables associated with the section B (QoLA) questionnaire scores as perceived by caregivers.

		Univariate Regression Analysis				Multivariate Regression Analysis		
Predictors	Estimate	95% Confidence Interval		*p*-Value	Estimate	95% Confidence Interval		*p*-Value
		Lower Bound	Upper Bound			Lower Bound	Upper Bound	
Age								
30–<40 vs. 20–<30	0.661	−4.216	5.539	0.790	−8.465	−8.792	−8.138	<0.001
40+ vs. 20–<30	−6.246	−10.906	−1.586	0.009	−34.203	−34.605	−33.802	<0.001
Marital Status								
Divorced/Widowed—married	18.657	14.702	22.613	<0.001	25.045	24.587	25.503	<0.001
Education								
University school—Read and write	−4.428	−8.769	−0.088	0.046	-	-	-	-
Secondary school—Read and write	−2.513	−6.702	1.676	0.238	-	-	-	-
Income								
Enough—Not enough	−4.577	−7.775	−1.379	0.005	−11.699	−12.101	−11.297	<0.001
Occupation								
Working—housewife	14.865	8.811	20.919	<0.001	17.202	16.733	17.671	<0.001
No. of children								
4–6 vs. 1–3	8.525	5.210	11.840	<0.001	7.217	6.753	7.680	<0.001
>6 vs. 1–3	−0.617	−5.369	4.136	0.798	30.272	29.860	30.683	<0.001
Children with special needs								
Two—one	−1.257	−4.813	2.299	0.487	-	-	-	-
Caregiver training								
Yes—no	−3.036	−6.925	0.853	0.125	−5.086	−5.487	−4.685	<0.001
Medical diseases								
Yes—no	3.639	0.139	7.139	0.042	12.377	12.012	12.743	<0.001
Another person helped in caring for the child.								
Yes—no	4.218	0.399	8.037	0.031	30.395	29.870	30.920	<0.001

**Table 7:** Correlation between Total QoL and child difficulties scores as perceived by caregivers in the (QoLA) questionnaire.

	Total Caregiver QoL (Section A)	
	r	*p*-Value
Total child difficulties (section B)	−0.415	0.000 **

** highly statistically significant *p* < 0.01.

## Data Availability

The data that support the findings of this study are available from the corresponding author upon reasonable request.

## References

[1] Hodges H., Fealko C., Soares N. (2020). Autism spectrum disorder: Definition, epidemiology, causes, and clinical evaluation. Transl. Pediatr..

[2] Al-Beltagi M. (2021). Autism medical comorbidities. World J. Clin. Pediatr..

[3] CDC (2022). Data and Statistics on Autism Spectrum Disorder. https://www.cdc.gov/autism/data-research/index.html.

[4] Salhi H.O., Al-Nasser L.A., Taher L.S., Al-Khathaami A.M., El-Metwally A.A. (2014). Systemic review of the epidemiology of autism in Arab Gulf countries. Neurosciences.

[5] Alenezi S., Alkhiri A., Hassanin W., AlHarbi A., Al Assaf M., Alzunaydi N., Alsharif S., Alhaidar M., Alnujide A., Alkathiri F. (2022). Findings of a Multidisciplinary Assessment of Children Referred for Possible Neurodevelopmental Disorders: Insights from a Retrospective Chart Review Study. Behav. Sci..

[6] Ibrahimagic A., Patkovic N., Radic B., Hadzic S. (2021). Communication and language skills of autistic spectrum disorders in children and their parents’ emotions. Mater. Socio Medica.

[7] Kheir N.M., Ghoneim O.M., Sandridge A.L., Hayder S.A., Al-Ismail M.S., Al-Rawi F. (2012). Concerns and considerations among caregivers of a child with autism in Qatar. BMC Res. Notes.

[8] Alshahrani M.S., Algashmari H. (2021). The moderating effect of financial stress and autism severity on development of depression among parents and caregivers of Autistic children in Taif, Saudi Arabia. J. Fam. Med. Prim. Care.

[9] Alhazmi A., Petersen R., Donald K.A. (2018). Quality of life among parents of South African children with autism spectrum disorder. Acta Neuropsychiatr..

[10] Nik Adib N.A., Ibrahim M.I., Ab Rahman A., Bakar R.S., Yahaya N.A., Hussin S., Wan Mansor W.N.A. (2019). Predictors of caregivers’ satisfaction with the management of children with autism spectrum disorder: A study at multiple levels of health care. Int. J. Environ. Res. Public Health.

[11] Saito A., Stickley A., Haraguchi H., Takahashi H., Ishitobi M., Kamio Y. (2017). Association between autistic traits in preschool children and later emotional/behavioral outcomes. J. Autism Dev. Disord..

[12] Eapen V., Guan J. (2016). Parental quality of life in autism spectrum disorder: Current status and future directions. Acta Psychopathol..

[13] Alamri D.A., Mahzari Q.A., Shaqran T., Albalawi J.A., Alanazi F.K., Rheab (2020). Impact of autism on parents’ and caregivers’ quality of life in Tabuk. Int. J. Med. Res. Health Sci..

[14] Alenazi D.S., Hammad S.M., Mohamed A.E. (2020). Effect of autism on parental quality of life in Arar city, Saudi Arabia. J. Fam. Community Med..

[15] Al-Jabri B.A., Abualhamael R.M., Al Hazza M.T., Bahabri S.A., Alamri Y.M., Alghamdi B.M. (2022). Quality of life of caregivers of autistic children in Saudi Arabia: Cross-sectional study. Neurosciences.

[16] Gobrial E. (2018). The lived experiences of mothers of children with the autism spectrum disorders in Egypt. Soc. Sci..

[17] Al-Dakroury W.A., Alnemary F.M., Alnemary F. (2022). Autism in the Kingdom of Saudi Arabia: Current situation and future perspectives for services and research. Perspect. ASHA Spec. Interest Groups.

[18] Saudi Arabia (2018). Vision 2030 Kingdom of Saudi Arabia Quality of Life Program 2020. https://www.vision2030.gov.sa/en/explore/programs/quality-of-life-program.

[19] Eapen V., Črnčec R., Walter A., Tay K.P. (2014). Conceptualization and development of a quality of life measure for parents of children with autism spectrum disorder. Autism Res. Treat..

[20] Faul F., Erdfelder E., Lang A.G., Buchner A. (2007). G* Power 3: A flexible statistical power analysis program for the social, behavioral, and biomedical sciences. Behav. Res. Methods.

[21] McConachie H., Mason D., Parr J.R., Garland D., Wilson C., Rodgers J. (2018). Enhancing the validity of a quality of life measure for autistic people. J. Autism Dev. Disord..

[22] Lord C., Brugha T.S., Charman T., Cusack J., Dumas G., Frazier T., Jones E.J.H., Jones R.M., Pickles A., State M.W. (2020). Autism spectrum disorder. Nat. Rev. Dis. Prim..

[23] Dey M., Paz Castro R., Haug S., Schaub M.P. (2019). Quality of life of parents of mentally-ill children: A systematic review and meta-analysis. Epidemiol. Psychiatr. Sci..

[24] Ni’matuzahroh Suen M.W., Ningrum V., Widayat Yuniardi M.S., Hasanati N., Wang J.H. (2022). The Association between parenting stress, positive Reappraisal Coping, and quality of life in parents with Autism Spectrum disorder (ASD) children: A systematic review. Healthcare.

[25] Pisula E., Porębowicz-Dörsmann A. (2017). Family functioning, parenting stress and quality of life in mothers and fathers of Polish children with high functioning autism or Asperger syndrome. PLoS ONE.

[26] Vasilopoulou E., Nisbet J. (2016). The quality of life of parents of children with autism spectrum disorder: A systematic review. Res. Autism Spectr. Disord..

[27] Salami S., Alhalal E. (2024). Gender differences in predictors of quality of life for parents of children with Autism Spectrum disorder in Saudi Arabia. J. Pediatr. Nurs..

[28] Eapen V., Karlov L., John J.R., Beneytez C., Grimes P.Z., Kang Y.Q., Mardare I., Minca D.G., Voicu L., Malek K.A. (2023). Quality of life in parents of autistic children: A transcultural perspective. Front. Psychol..

[29] Al-Lihabi A., Alharbi S.T., Alsheraimi R., Alharbi A.M., Alofi M., Azouni S., Albaidani A. (2025). Quality of Life and Sleep Among Parents of Children With Autism Spectrum Disorder in Medina. Cureus.

[30] Kheir N., Ghoneim O., Sandridge A.L., Al-Ismail M., Hayder S., Al-Rawi F. (2012). Quality of life of caregivers of children with autism in Qatar. Autism.

[31] Marsack-Topolewski C.N., Church H.L. (2019). Impact of caregiver burden on quality of life for parents of adult children with autism spectrum disorder. Am. J. Intellect. Dev. Disabil..

[32] Turnage D., Conner N. (2022). Quality of life of parents of children with Autism Spectrum Disorder: An integrative literature review. J. Spec. Pediatr. Nurs..

[33] Subke A.A., Khan A.A., Alharthi S.A. (2020). Factors affecting quality of life among parents having a child with autism spectrum disorder in Jeddah, 2019 (cross-sectional study). J. Prev. Med. Holist. Health.

[34] Duarte C.S., Bordin I.A., de Oliveira A., Bird H. (2003). The CBCL and the identification of children with autism and related conditions in Brazil: Pilot findings. J. Autism Dev. Disord..

[35] Kasem A., Abuhammad S., Jamal N. (2024). Parents of children with autism spectrum disorder quality of life in Jordan: A comparative study. Future Sci. OA.

[36] Dardas L.A., Ahmad M.M. (2014). Quality of life among parents of children with autistic disorder: A sample from the Arab world. Res. Dev. Disabil..

[37] Asahar S.F., Malek K.A., Isa M.R. (2021). Quality of Life and Child’s Autism-Specific Difficulties among Malaysian Main Caregivers: A Cross-Sectional Study. Int. J. Environ. Res. Public Health.

[38] Konowałek Ł., Kotowska-Bąbol M., Łukasik J., Remiszewski P., Wolańczyk T. (2025). Quality of life among parents of autistic children: Questionnaire validation study and multivariate analysis of associated factors. Front. Psychiatry.

[39] Chen X., Tong J., Wang X., Zhu L., Tao Y., Zhao H., Liu C., Wang Y., Zhao C., Yan D. (2025). Factors related to quality of life in caregivers of children with autism spectrum disorder: Emphasizing challenges in the context of Eastern China. BMC Public Health.

[40] Volgyesi-Molnar M., Gyori M., Eapen V., Borsos Z., Havasi A., Jakab Z., Janoch L., Nemeth V., Oszi T., Szekeres A. (2025). Quality of Life in Hungarian Parents of Autistic Individuals. J. Autism Dev. Disord..

[41] Samadi S.A., Ghanimi F., McConkey R. (2025). The Quality of Life of Iranian Mothers and Fathers of Children with Autism. Children.

[42] Rezq K.A., Albalawi H.M.H., Alharbi H.F. (2025). Exploring Social Support and Quality of Life Among Mothers of Children with Autism Spectrum Disorders: A Cross-Sectional Study. Healthcare.

[43] Alhazmi A., Alduraibi A., Alhemaid M., Albreakan A., Alshaqha R. (2023). Quality of Life among the Parents of Saudi Arabian children with Autistic Spectrum Disorder, Riyadh, KSA. Middle East J. Fam. Med..

[44] Patel A.D., Arya A., Agarwal V., Gupta P.K., Agarwal M. (2022). Burden of care and quality of life in caregivers of children and adolescents with autism spectrum disorder. Asian J. Psychiatr..

[45] Alkhateeb J.M., Hadidi M.S., Mounzer W. (2022). The Impact of Autism Spectrum Disorder on Parents in Arab Countries: A Systematic Literature Review. Front. Psychol..

